# Characterization of Three Intermediates in an Unusual Copper-Dependent Enzyme

**DOI:** 10.1021/acscentsci.5c00732

**Published:** 2025-05-06

**Authors:** Marcel Swart, Isaac Garcia-Bosch

**Affiliations:** † ICREA, Pg. Lluís Companys 23, 08010 Barcelona, Spain; ‡ IQCC and Dept. Chemistry, 16738 Universitat de Girona, Campus Montilivi, 17003 Girona, Spain; § Department of Chemistry, 6612 Carnegie Mellon University, Pittsburgh, Pennsylvania 15213, United States

## Abstract

Three intermediates in the formylglycine-generating enzyme are characterized at the molecular level.

## Graphical abstract

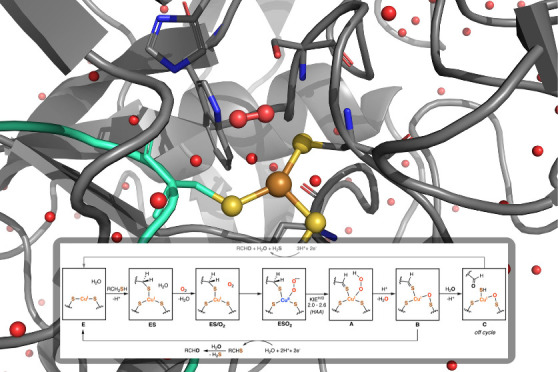

The activation of small molecules like methane, dinitrogen, or dioxygen in enzymatic and/or homogeneous catalysis typically requires the involvement of (transition) metals like iron, nickel, or copper. These metals enable the controlled transfer of electrons to, for example, O_2_. Nevertheless, when copper is involved, sometimes ‘Cu-rious’ chemistry takes place. In this issue of *ACS Central Science*, Bertozzi, Solomon, and co-workers report^1^ their latest findings on one of the more exotic examples of a copper-dependent enzyme.

Initially, it was not even known that the enzyme required copper, nor what the mechanism was for the reaction taking place, which transforms a cysteine in a **C**XPXR sequence in the substrate into the corresponding (formylglycine) aldehyde (see Figure 1A). That all changed in 2015 when two groups independently observed^2,3^ a substantial rate enhancement when copper is bound in the active site. Since then there have been reports combining spectroscopy and crystal structures of the enzyme with silver,^4^ copper,^5^ copper plus dioxygen,^6^ and copper and substrate,^7^ complemented by a recent quantum mechanics/molecular mechanics (QM/MM) study^8^ on the electronic structure of the intermediates. Interestingly, the substrate binds covalently to copper through its cysteine, leading to a trigonal coordination of cysteines surrounding the Cu­(I) in the active site (see species **E** and **ES** in Figure 1B).

**Figure 1:**
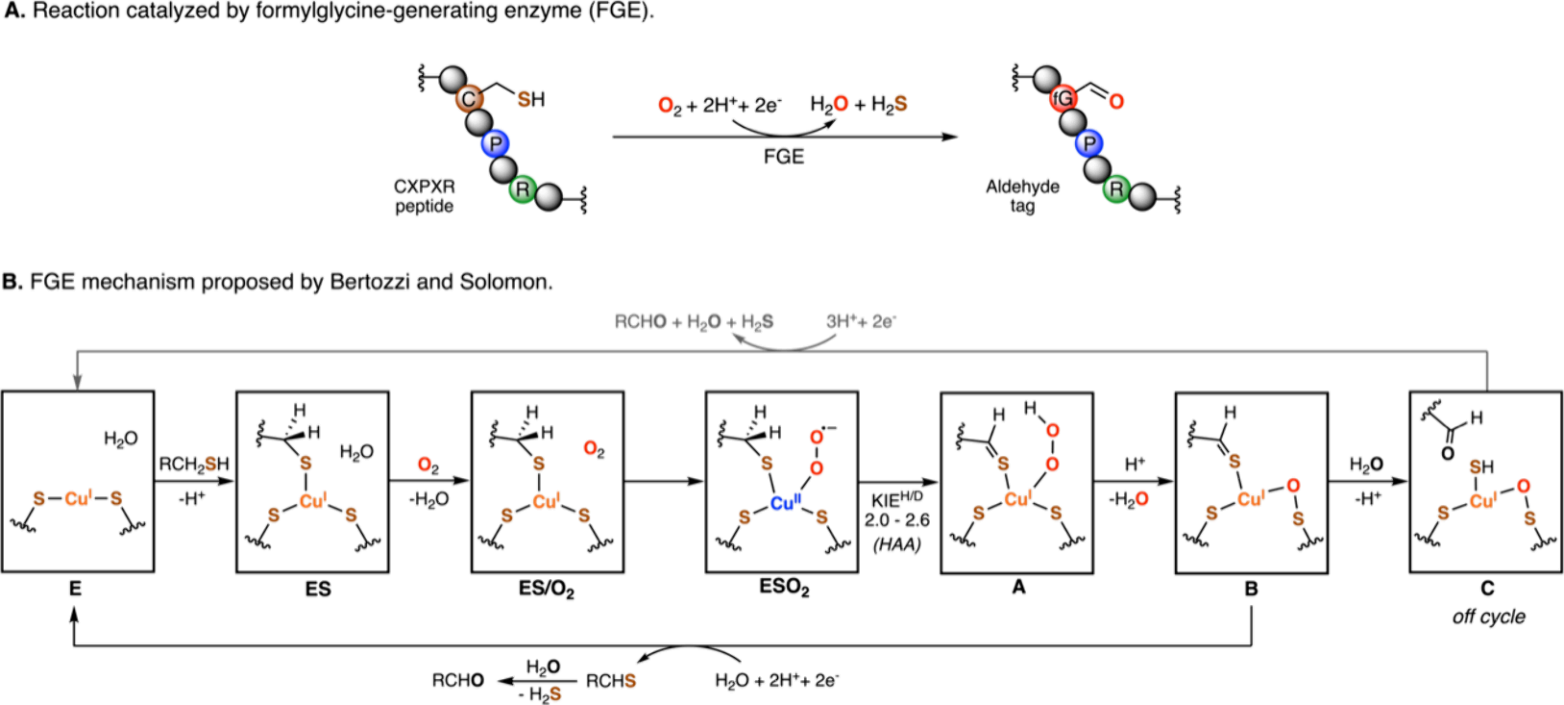
Overview of the reaction mechanism of the FGE enzyme and the intermediates in it that have been characterized in ref 1.

Apart from the unusual Cys_3_-bound Cu­(I) in the active site, this formylglycine-generating enzyme (FGE) is special within bioinorganic chemistry because it involves a rare mononuclear copper active site, without any additional cofactor, which provides half of the four electrons (and four protons) needed to transform dioxygen into product and water. The other two electrons are supplied by an unknown natural external reductant (in the experiments with the FGE enzyme included in this article, the 2e-reductant dithiothreitol (DTT) was used). Different pathways have been proposed, all of which start from an unusual intermediate where dioxygen is **not** activated through an inner-sphere mechanism by coordinating to copper. Instead, it binds remotely from copper­(I) at a distance of ca. 3.1 Å in the resting state (species **ES/O**_**2**_ in Figure 1B).^6^ A Gibbs free energy barrier of ca. 3 kcal·mol^–1^ needs to be surpassed^8^ to generate the Cu­(II)-superoxide species (**ESO**_**2**_, Figure 1B) in an endergonic process; the back reaction has an even smaller barrier of ca. 0.5 kcal·mol^–1^, highlighting the thermal instability of the copper superoxide, unlike in other copper enzymes. However, the copper-superoxide performs the first step in the mechanism with a sizable barrier (18.8 kcal·mol^–1^ relative to **ES/O**_**2**_, or 16.4 kcal·mol^–1^ relative to **ESO**_**2**_): the hydrogen-atom abstraction of the pro-(R)-β-hydrogen atom of the substrate cysteine residue.

The start of the reaction cycle by this hydrogen-atom abstraction was already inferred and demonstrated by QM/MM calculations,^8^ but the subsequent steps were speculative. In the current contribution Bertozzi, Solomon, and co-workers used UV–vis spectroscopy in combination with stopped-flow to determine the kinetics of the reaction steps.

After reacting **ES** with O_2_, three intermediates were observed (intermediates **A**, **B**, and **C**), which were further characterized by electron paramagnetic resonance (EPR) and extended X-ray absorption fine structure (EXAFS) spectroscopy, and (TD-)­DFT calculations.

Based on kinetic isotope effect experiments, intermediate **A** was proposed to be the species formed right after hydrogen atom transfer from the substrate to the putative Cu-superoxide. EPR and EXAFS indicated that intermediate **A** was a diamagnetic cuprous species, formulated as a Cu­(I)-hydroperoxide bound to a peptidyl-thioaldehyde (see Figure 1B). Using the same approach, the authors assigned intermediate **B** as a Cu­(I)-sulfenate-thioaldehyde complex (in which one of the oxygens from hydroperoxo is inserted into one of the Cu–S bonds to the enzyme, and the other cleaved off as water) and intermediate **C** as a Cu­(I)-sulfenate-HS^–^ species (derived from the hydrolysis of the peptidyl-thioaldehyde). The authors noted that intermediate **C** was formed slowly and in the absence of reductant, which suggested that it might not be part of the catalytic cycle of FGE. Hence, the catalytic cycle is most likely closed via reduction of intermediate **B**, leading to the starting cuprous bis-thiolate form of the enzyme (**E**) that is then ready for the next cycle.

Although three intermediates in the catalytic cycle have now been characterized, there are still details of the mechanism that leave questions to be answered in future studies. Where does the oxygen of the aldehyde come from?

Is it one of the oxygens from dioxygen or is it coming from water? And if it is the latter, is it the water molecule generated during the catalytic cycle, or is it external water? Isotope labeling experiments, in combination with resonance Raman spectroscopy, might shed light on this issue. In the “consensus” mechanism (reinforced by the contribution of Bertozzi, Solomon, and co-workers) the aldehyde’s oxygen comes from water, albeit with the caveat that there is yet no evidence whether the oxygen comes from external water or from the water molecule generated during the catalytic cycle. Note that Shaik, Lai, and co-workers proposed instead that the aldehyde oxygen is coming from dioxygen (Figure 2): they observed in their QM/MM calculations that after the hydrogen atom abstraction, a Cu­(II) hydroperoxo species is formed (as also suggested by Tainer, Solomon, Bertozzi, and co-workers in a previous study^5^). The distal oxygen could then attack the generated carbon radical on cysteine to form a Cu­(I)-alkylperoxo species, which after cleavage of the O–O bond would lead to the aldehyde (based on an oxygen coming from dioxygen).

**Figure 2:**
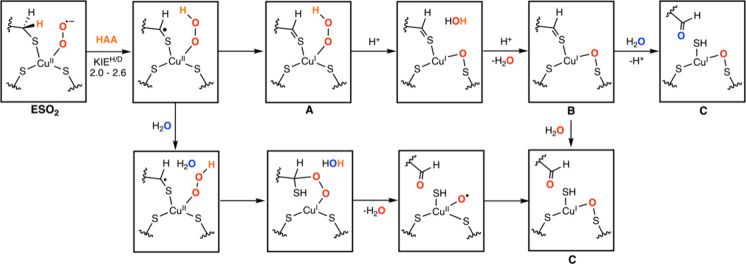
Alternative pathways^8^ that might be present in the reaction mechanism, highlighting the role of either water (external in blue, or generated in the catalytic cycle in red) or dioxygen O atoms for the generation of the aldehyde.

Interest in the mechanism of the FGE enzyme goes beyond scientific curiosity and the Cu-rious bioinorganic aspects.

There is now ample evidence to suggest that any cysteine of a **C**XPXR sequence in a substrate will be transformed in the corresponding formylglycine aldehyde (fGly).

This promiscuity opens up a path to biotechnology and therapeutic applications, since the formed fGly is rare among the amino acid library from which proteins are made. This makes fGly an excellent target for bioorthogonal conjugation^2^ in recombinant proteins.

Similarly, the unusual coordination chemistry of a Cys_3_-bound Cu­(I), with a lingering dioxygen bound remotely from it in the active site pocket, together with the unusual coordination environments of copper in the intermediates of the catalytic cycle provides a unique opportunity (and challenge) for synthetic bioinorganic chemists to synthesize bioinspired copper complexes that mimic its coordination chemistry.

If successful, spectroscopic and/or structural characterization would provide additional evidence for the reaction mechanism that determines the catalytic cycle, and the ‘Cu-rious’ chemistry contained within it.

## References

[1] Kipouros I., Lim H., Appel M.J., Meier K.K., Hedman B., Hodgson K.O., Bertozzi C.R., Solomon E.I. (2025). Mechanism of O_2_ Activation
and Cysteine Oxidation by the Unusual Mononuclear Cu­(I) Active Site
of the Formylglycine-Generating Enzyme. ACS
Cent. Sci..

[2] Holder P. G., Jones L. C., Drake P. M., Barfield R. M., Bañas S., de Hart G. W., Baker J., Rabuka D. (2015). Reconstitution of Formylglycine-generating
Enzyme with Copper­(II) for Aldehyde Tag Conversion. J. Biol. Chem..

[3] Knop M., Engi P., Lemnaru R., Seebeck F. P. (2015). In Vitro Reconstitution
of Formylglycine-Generating Enzymes Requires Copper­(I). ChemBioChem..

[4] Meury M., Knop M., Seebeck F. P. (2017). Structural Basis for Copper-Oxygen
Mediated C-H Bond Activation by the Formylglycine-Generating Enzyme. Angew. Chem., Int. Ed..

[5] Appel M. J., Meier K. K., Lafrance-Vanasse J., Lim H., Tsai C., Hedman B., Hodgson K. O., Tainer J. A., Solomon E. I., Bertozzi C. R. (2019). Formylglycine-generating enzyme binds substrate directly
at a mononuclear Cu­(I) center to initiate O_2_ activation. Proc. Natl. Acad. Sci. U.S.A..

[6] Leisinger F., Miarzlou D. A., Seebeck F. P. (2021). Non-Coordinative
Binding of O_2_ at the Active Center of a Copper-Dependent
Enzyme. Angew. Chem., Int. Ed..

[7] Miarzlou D. A., Leisinger F., Joss D., Häussinger D., Seebeck F. P. (2019). Structure of formylglycine-generating
enzyme in complex
with copper and a substrate reveals an acidic pocket for binding and
activation of molecular oxygen. Chem. Sci..

[8] Wu Y., Zhao C., Su Y., Shaik S., Lai W. (2023). Mechanistic
Insight into Peptidyl-Cysteine Oxidation by the Copper-Dependent Formylglycine-Generating
Enzyme. Angew. Chem., Int. Ed..

